# Cyclic Strain-Induced Cytoskeletal Rearrangement of Human Periodontal Ligament Cells via the Rho Signaling Pathway

**DOI:** 10.1371/journal.pone.0091580

**Published:** 2014-03-11

**Authors:** Jinsong Pan, Tingle Wang, Li Wang, Wantao Chen, Meng Song

**Affiliations:** 1 Department of Oral and Maxillofacial Surgery, Ninth People’s Hospital, Shanghai Jiao Tong University School of Medicine, Shanghai Key Laboratory of Stomatology and Shanghai Research Institute of Stomatology, Shanghai, China; 2 Department of Stomatology, Central Hospital of Minhang District, Shanghai, China; 3 Department of Stomatology, First People’s Hospital, Shanghai Jiao Tong University School of Medicine, Shanghai, China; Casey Eye Institute, United States of America

## Abstract

**Background:**

Although mechanical stimulations are known have a significant impact on cytoskeletal rearrangement, little is known regarding the behavioral alteration of human periodontal ligament cells (hPDLCs) under cyclic strain. The aim of this study was to elucidate the role of the Rho signaling pathway on cyclic strain-induced cytoskeletal rearrangement of hPDLCs.

**Methods:**

Healthy hPDLCs obtained from teeth extracted for orthodontic purposes were subjected to cyclic strain with physiological loading (10%) at a frequency of 0.1 Hz for 6 h or 24 h using a FX-5000T system. Changes in cell morphology were examined by phase-contrast microscopy, while F-actin reorganization was observed by phalloidin staining and confocal microscopy. Protein expression was analyzed through western blot analysis.

**Results:**

Significant enhancement of cytoskeletal reorganization was observed following exposure to the cyclic strain. In addition, a significant increase was noted in the expression levels of GTP-Rho, Rho-associated protein kinase (ROCK) and p-cofilin, whereas the expression levels of Rho GDP dissociation inhibitors alpha (Rho-GDIa) were reduced in the hPDLCs, compared with the static control cells. More importantly, the Rock inhibitor Y-27632 suppressed cyclic strain-induced cytoskeletal rearrangement of hPDLCs. Additionally, Y-27632 and overexpression of Rho-GDIa were found to lower p-cofilin protein expressions under cyclic strain, while Rho-GDIa siRNA transfection had the opposite effect on the hPDLCs.

**Conclusion:**

Cyclic strain promotes cytoskeletal rearrangement of hPDLCs by downregulating the expression levels of Rho-GDIa and upregulating the expression levels of GTP-Rho, Rock and p-cofilin. These observations may provide valuable insight into understanding orthodontic tooth movement as well as alveolar bone remodeling.

## Introduction

Mechanical stimulation is an important factor of tissue remodeling, and *in vivo,* many cells, including fibroblasts, endothelial cells, and smooth muscle cells, are load-sensitive. They possess the ability to sense a mechanical load and change their diverse cellular functions, such as cell proliferation and extracellular matrix expression, resulting in alterations of structure, composition, and function of living tissues [1].

Mechanical force applied to a tooth can be transmitted to the periodontium surrounding the root and initiates remodeling activities that allow the tooth to move through alveolar bone. The periodontal ligament (PDL) is the soft connective tissue situated between the cementum covering the root of the tooth and the bone forming the socket wall. The PDL consists of cells surrounded by extracellular matrix comprised of fibers and ground substance. In addition to providing support to the tooth within its socket, the PDL compensates the forces generated by occlusion and mastication, aids in the development and maintenance of the periodontium, and serves as a sensory receptor [2].

Human periodontal ligament cells (hPDLCs), which are the main components of the PDL, are activated after injury or periodontal surgery, following which they proliferate, migrate into the wound site, and synthesize new matrix components until the defect has been corrected [3], [4]. Therefore, investigating the responses of hPDLCs to mechanical strain application may aid in understanding orthodontic tooth movement and alveolar bone remodeling.

The cytoskeleton is a complex organ in eukaryotic cells and has important functions such as aiding cell motility and maintaining cell morphology. Periodontal tissue is reconstructed during orthodontic tooth movement and the cytoskeleton of periodontal ligament cells changes accordingly, suggesting that cyclic strain plays an important role in the cytoskeleton. However, the exact mechanism of cyclic strain in the cytoskeleton of hPDLCs is not yet clearly understood.

Previous studies have shown that cyclic strain can evoke various intracellular signaling pathways, such as Rho family GTPases, mitogen-activated protein kinases (MAPKs), and PI3K/Akt [5]; however, the key signaling pathway of cyclic strain-induced hPDLC cytoskeletal rearrangement remains to be elucidated.

A previous research suggested that small GTPase Rho and its downstream effector can mediate cyclic strain-induced migration of vascular smooth muscle cells [6]. This mechanism is particularly interesting because Rho is a major organizer of the cytoskeleton [7] and can regulate the formation of actin stress fibers by activating Rho-associated protein kinase (ROCK), which phosphorylates, which in turn phosphorylates cofilin. Cofilin binds to both actin monomers and polymers, and promotes the disassembly of actin filaments, and this function is suppressed by its phosphorylation [8]. These observations led us to hypothesize that the Rho signaling pathway may play an important role during cyclic strain of hPDLCs, leading to cytoskeletal rearrangement and tooth movement.

In the present study, hPDLCs were subjected to cyclic strain with physiological loading (10%) [9], [10] to investigate cytoskeletal rearrangement of hPDLCs. Subsequently, the role of the Rho signaling pathway in cyclic strain-induced cytoskeletal rearrangement of hPDLCs was determined in order to understand the mechanism by which hPDLCs respond to mechanical strain.

## Materials and Methods

### Sample collection and ethics statement

The hPDLCs were obtained from orthodontically extracted healthy teeth belonging to different donors. Written informed consent was obtained from the parents of the donors, in accordance with the Declaration of Helsinki. The study protocol was approved by the Ethics Committee at Shanghai JiaoTong University (China).

### Antibodies and reagents

The specific inhibitor of ROCK (Y-27632) was obtained from Calbiochem (10 μM, La Jolla, CA). Antibodies used in western blot and immunocytochemistry experiments were as follows: monoclonal anti-cytokeratin (D1E4), monoclonal anti-vimentin (D21H3), polyclonal anti-phospho-cofilin, polyclonal anti-cofilin (1∶500 from Cell Signaling, Beverly, MA), and anti-ROCK1(sc-17794), anti-RhoA(sc-179) (1∶500 from Santa Cruz Biotechnology, Santa Cruz, CA, USA), and polyclonal anti-RhoGDIα antibody (1∶500 Sigma-Aldrich, St. Louis, MO). FITC-labeled phalloidin was purchased from Molecular Probes (1∶100 Eugene, OR).

### Cell Culture and treatment

The hPDLCs were isolated by enzymatic digestion of PDL from healthy teeth following orthodontic extraction and were cultured in Dulbecco’s modified Eagle’s medium (DMEM, Gibco-BRL, Grand Island, NY) supplemented with 10% fetal bovine serum (FBS), penicillin (100 units/ml), and streptomycin (100 units/ml). Cells were incubated at 37°C in a humidified atmosphere of 5% CO_2_, and those between passages 4–8 were used in all experiments. The fourth passage cells were stained with vimentin and cytokeratin antibodies for characterization (Fig. S1).

For cyclic strain loading experiments, hPDLCs were seeded onto collagen I-coated 6-well Bioflex plates (Flexcell International, USA) at a density of 3×10^5^ cells/well. Cells achieving 95% confluence were serum starved in DMEM for 24 h and then subjected to cyclic strain using a Flexercell Tension Plus system (FX-5000T, Flexcell International, USA) with a magnitude of 10% elongation at a frequency of 0.1 Hz for 6 h or 24 h.

### Cytoskeletal staining

The cells were cultured in collagen I-coated, 6-well Bioflex plates containing serum-free medium for 24 h and then stimulated with cyclic strain for up to 1 day. At 6 h and 24 h, the cells were rinsed in phosphate-buffered saline (PBS) for 3 min, fixed in 5% paraformaldehyde (PFA) for 30 min, and permeabilized with 0.1% Triton in PBS for 10 min. Then, the cells were incubated with FITC-labeled phalloidin diluted at 1∶100 at room temperature. Finally, the cells were stained with DAPI for 5 min (1∶200 dilution) at room temperature. After being washed with PBS, cells were mounted with fluorSave reagent (Calbiochem) and analyzed by confocal microscopy (Leica TCS 4D). Three randomly selective microscopic fields were analyzed.

### Western blot analysis

Cells from experimental and control groups were scraped from the 6-well plates and placed into 300 μl of ice-cold lysis buffer [50 mM Tris–HCl (pH∶7.4), 150 mM NaCl, 1 mM EDTA, 1% NP-40, 0.5% Na-deoxycholate, and 20 μl/ml protease inhibitor cocktail (Pharmingen BD Biosciences, San Jose, CA)]. The samples were clarified by centrifugation at 13,000 rpm for 5 min at 4°C and boiled for 5 min with Laemmli sample buffer containing 100 mM NaF. Protein concentrations were determined by the Bradford method (Bio-Rad Laboratories, Richmond, CA). Equivalent protein amounts were separated on 10% SDS–polyacrylamide gels and transferred to Immobilon-P polyvinylidene fluoride membranes (Millipore Corporation, Bedford, MA). The blots were then hybridized with specific primary antibodies and secondary antibodies labeled with IR Dyes. Signals were observed using an Odyssey Infrared Imaging System (LI-COR Biosciences, Lincoln, NE, USA).

### Activated rhoA western blot analysis

A total of 5×10^6^ cell lysates from experimental and control groups were incubated with Rhotekin-RBD (Rho binding domain) bound to glutathion-agarose beads (Upstate Biotechnology) for 45 min at 48°C. After washing three times with MLB, reduced SDS sample buffer 5×(containing 40 mM dithiothreitol) was added to the beads, which were subsequently heated at 95°C for 5 min. After centrifugation, precipitated GTP-RhoA samples were loaded onto 15% SDS–poly-acrylamide gel, subjected to electrophoresis and transferred to nitrocellulose membrane (Hybond ECL,Amersham Biosciences,Upsala,Sweden).The membrane was then incubated for 45 min under constant agitation, in 0.1% Tween 20-TBS solution (T-TBS) containing 5% dry fat milk, washed three times in T-TBS and exposed to rabbit anti-RhoA polyclonal antibody (1∶100 in 1%dry fat milk T-TBS; Santa Cruz Biotechnology) overnight at 48°C. Three washings with T-TBS were followed by 30-min incubation with anti-rabbit HRP conjugated antibody (1∶10,000 in T-TBS, Sigma), then five washings with T-TBS and one more with bi-distilled water. Detection was performed by enhanced chemiluminescence (ECL,Amersham Biosciences). All steps were performed at room temperature, unless otherwise specified. Negative controls were performed by using blocked primary antibody. A negative control for RhoA pull-down was also performed by loading beads only.

### RNA Interference and Inhibitor treatment

The mRNA sequences of human Rho-GDIa were acquired from NCBI GenBank. Small interfering RNAs (siRNAs) against human Rho-GDIa was designed and synthesized by GenePharma Biological Company (Shanghai, P.R. China). The sequences of small interfering RNAs (siRNA) targeting Rho-GDIa were as follows: 5′-GAGAUAGUGUCCGGCAUGAdTdT-3′ and 5′-UCAUGCCGGACACUAUCUCdTdT-3′.

Subsequent to seeding for 24 h, the cells were transfected with siRNA by Lipofectamine™ 2000 (Invitrogen) at a final RNA concentration of 100 nM according to the manufacturer’s instructions. After incubation at 37°C for 6 h in a humidified CO_2_ incubator, the transfected medium was replaced with DMEM for 18 h prior to cyclic strain. Nonsilencing siRNA that does not recognize any known homology to human genes was used as a negative control (NC).

For inhibitor studies, hPDLCs were preincubated with Y-27632 (10 μM) [11], [12], a specific Rock inhibitor, for 2 h before cyclic strain application. The hPDLCs without any treatment under the same conditions were used as the control.

### Plasmid construction

The eukaryotic expression plasmid pcDNA 3.0/Rho-GDIa was constructed as described in our previous report [13]. Total RNA from hPDLCs was prepared with Trizol reagent and reverse transcribed to cDNA, and the open-reading frame of Rho-GDIa was amplified and cloned into pcDNA3.0 vector. pcDNA 3.0/Rho-GDIa was verified by sequencing analysis.

### Cell transfection

Transfection of cells was performed with Lipofectamine™ 2000 reagent as described in our previous report [13]. Briefly, the cells were seeded in six-well flexcell plates at 90% confluence the day before transfection. The cells were transfected with pcDNA 3.0/Rho-GDIa by Lipofectamine™ 2000 according to the manufacturer’s instructions. After incubation at 37°C for 6 h in a humidified CO_2_ incubator, the transfected medium was replaced with DMEM for 18 h prior to cyclic strain. The null-plasmid transfection group was used as a negative control (Mock).

### Statistical analysis

Data were presented as the mean ± standard deviation (s.d.) of three separate experiments, and one-way ANOVA with Student–Newman–Keul’s test comparison was used for statistical significance with P ≤ 0.05.

## Results

### Effect of cyclic strain on the expression levels of Rho signaling pathway proteins and on cytoskeletal rearrangement of hPDLCs

**Figure 1:**
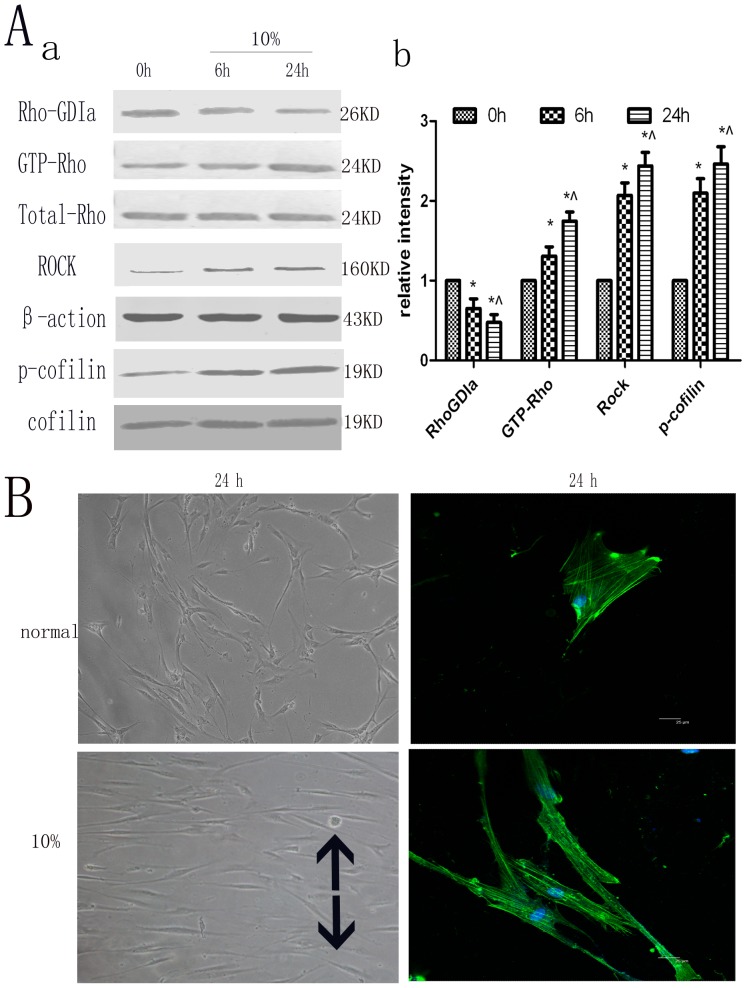
The effects of cyclic strain on the expression of Rho signaling pathway proteins and on cytoskeletal rearrangement of hPDLCs. (A) Effects of cyclic strain on Rho-GDIa, GTP-Rho, Rock and p-cofilin protein expression. Cells under cyclic strain of 10% physiological loading had Rho-GDIa protein expression levels that were significantly less than those in the static control, while the expression levels of GTP-Rho, Rock and phospho-cofilin were increased compared to those in the static control. Values shown are the mean±SD for each group from three independent experiments. * p<0.05 vs. control group, ∧p<0.05 vs. cyclic strain (6h). All the band intensities are firstly normalized to the loading controls then normalized to the 0h treatment and loading control ratio (B) Effects on cell morphology and cytoskeletal rearrangement as seen with cytoskeleton staining. The unstimulated control cells contained randomly oriented cytoplasmic fibers, while the cyclic strain-treated cells were gradually arranged parallel to each other, perpendicular to the direction of the tension force, with elongated cell bodies. The black arrows show the direction of stretching (Cell morphology original magnifications×200, Cytoskeletal rearrangement Bar: 25 μm).

The hPDLCs were subjected to cyclic strain (10% physiological loading) at 0.1 Hz frequency for 6 h or 24 h, during which the expression levels of the Rho-GDIa proteins were found to be significantly less than those in 0h treatment, while the expression levels of GTP-Rho, Rock, and phospho-cofilin were increased under cyclic strain when compared to the 0h treatment over a period of time (Fig. 1A).

The unstimulated control hPDLCs contained randomly oriented cytoplasmic fibers, whereas the cyclic strain-treated cells were gradually arranged parallel to each other, perpendicular to the direction of the tension force, and demonstrated cell body elongation (Fig. 1B).

### Rock-specific inhibitor Y-27632 and its effect on cytoskeletal rearrangement of hPDLCs

**Figure 2:**
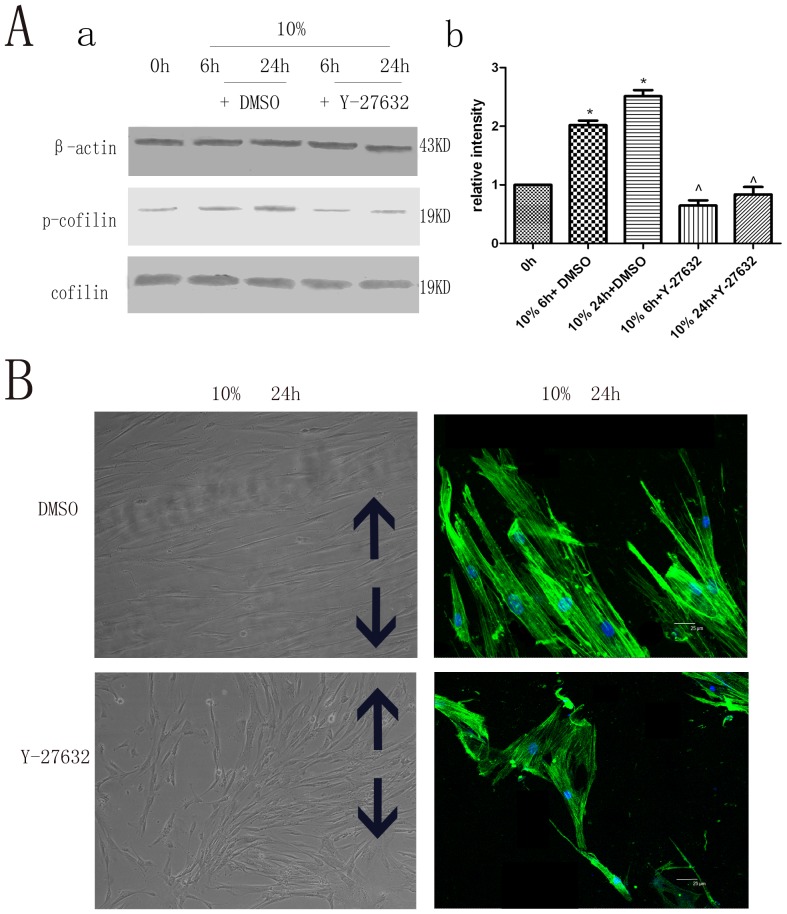
Rock-specific inhibitor Y-27632 and its effect on hPDLC cytoskeletal rearrangement. (A) Rock-specific inhibitor Y-27632 on p-cofilin protein expression. The expression levels of phospho-cofilin were less than those of the control group. Values shown are the mean±SD for each group from three independent experiments. * p<0.05 vs. control group, ∧p<0.05 vs. the hPDLCs treated with DMSO under the same conditions, respectively. All the band intensities are firstly normalized to the loading controls then normalized to the 0h treatment and loading control ratio.(B) Effects on cell morphology and cytoskeletal rearrangement by cytoskeleton staining. The cytoskeletal rearrangement of hPDLCs of the Y-27632 group was less than that of the control group. The black arrows show the direction of stretching (Cell morphology original magnifications×200, Cytoskeletal rearrangement Bar: 25 μm).

The cells were treated with the Rock-specific inhibitor Y-27632 2 h prior to cyclic strain application. Decreased expression of cofilin phosphorylation was observed compared to the control group (Fig. 2A). In addition, phase-contrast microscopy, immunocytochemistry, and confocal microscopy also revealed decreased cytoskeletal rearrangement of hPDLCs with the Rock-specific inhibitor (Fig. 2B), suggesting that inhibiting Rock may have contributed to the decreased cytoskeletal rearrangement of hPDLCs.

### Rho-GDP dissociation inhibitor alpha and its effect on other proteins in the Rho signaling pathway and cytoskeletal rearrangement of hPDLCs

**Figure 3:**
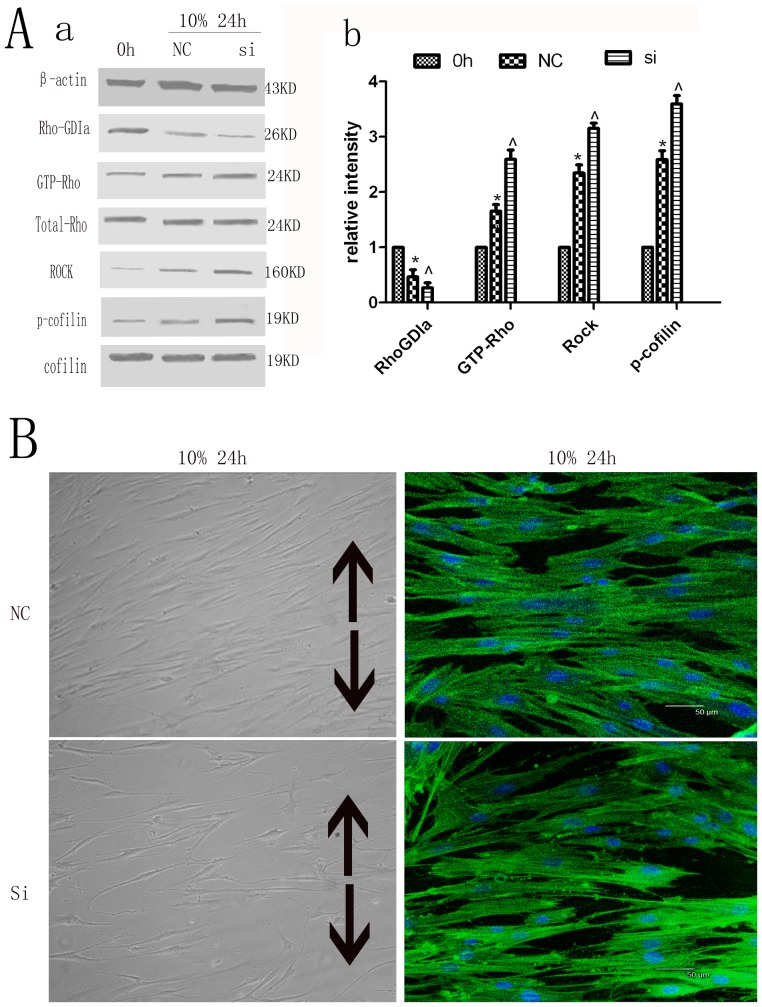
Silencing of Rho-GDP dissociation inhibitor alpha and its effect on the expression of Rho signaling pathway proteins. Silencing of Rho-GDP dissociation inhibitor alpha on Rho-GDIa, GTP-Rho, Rock, p-cofilin protein expression. The protein expression levels of Rho-GDIa in the Rho-GDIa siRNA transfection group were significantly less than those in the negative control (NC) group, while the expressions of GTP-Rho, Rock and phospho-cofilin were increased compared to the NC group. Values shown are the mean±SD for each group from three independent experiments. * p<0.05 vs. control group, ∧p<0.05 vs. NC group. All the band intensities are firstly normalized to the loading controls then normalized to the 0h treatment and loading control ratio. (B) Effects on cell morphology and cytoskeletal rearrangement by cytoskeleton staining. The cytoskeletal rearrangement of hPDLCs of Rho-GDIa siRNA group was more than that of the NC group. The black arrows show the direction of stretching (Cell morphology original magnifications×200, Cytoskeletal rearrangement Bar: 50 μm).

The protein expression levels of Rho-GDIa in the Rho-GDIa siRNA transfection group were significantly less than those in the negative control (NC) group in cultured unstimulated cells (Fig. S2). After subjection to cyclic strain for 24 h, the Rho-GDIa siRNA-treated cells exhibited decreased expression of Rho-GDIa, which was associated with significantly enhanced expression levels of GTP-Rho, Rock as well as cofilin phosphorylation when compared with the nonsilencing Rho-GDIa control cells (Fig. 3A). In addition, the cytoskeletal rearrangement of hPDLCs of Rho-GDIa siRNA group was more than that of the NC group (Fig. 3B).

### Overexpression of Rho-GDP dissociation inhibitor alpha and its effects on other proteins in the Rho signaling pahway and cytoskeletal rearrangement of hPDLCs

**Figure 4:**
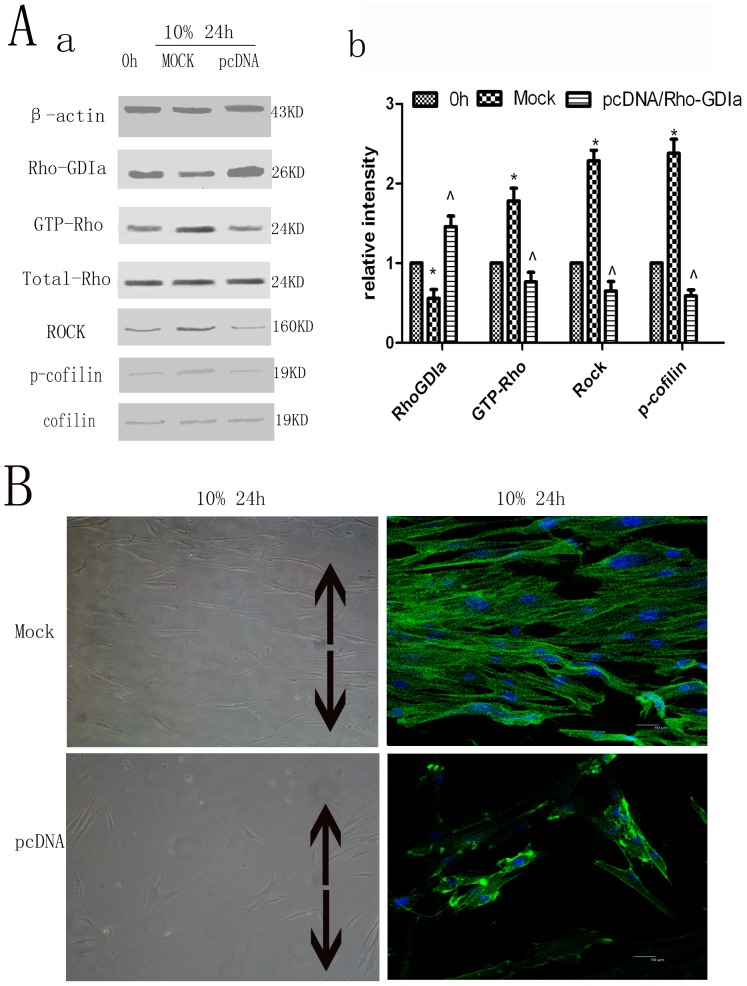
Overexpression of Rho-GDP dissociation inhibitor alpha and its effects on the expression of Rho signaling pathway proteins. Overexpression of Rho-GDP dissociation inhibitor alpha on Rho-GDIa, GTP-Rho, Rock and p-cofilin protein expression. The protein expression levels of Rho-GDIa after transfection with the pcDNA/Rho-GDIa group were significantly greater than those in the Mock group, while the expression levels of GTP-Rho, Rock and phospho-cofilin were less than those in the Mock group. Values shown are the mean±SD for each group from three independent experiments. * p<0.05 vs. control group, ∧p<0.05 vs. Mock group. All the band intensities are firstly normalized to the loading controls then normalized to the 0h treatment and loading control ratio. (B) Effects on cell morphology and cytoskeletal rearrangement by cytoskeleton staining. The cytoskeletal rearrangement of hPDLCs with transfection group was less than that of the Mock group. The black arrows show the direction of stretching (Cell morphology original magnifications×200, Cytoskeletal rearrangement Bar: 50 μm).

The protein expression levels of Rho-GDIa in cells overexpressing Rho-GDIa group were significantly more than those in the Mock group in cultured unstimulated cells (Fig. S3). After subjection to cyclic strain for 24 h, cells overexpressing Rho-GDIa demonstrated significantly decreased expression levels of GTP-Rho, Rock and cofilin phosphorylation when compared to the control cells that did not overexpress Rho-GDIa (Fig. 4A). In addition, the cytoskeletal rearrangement of hPDLCs overexpressing Rho-GDIa group was less than that of the Mock group (Fig. 4B).

## Discussion

Cyclic strain has been shown to influence many aspects of cellular metabolism that may subsequently affect other biological cell behaviors, such as proliferation, migration, and cytoskeletal arrangement, as well as the expressions of genes and proteins [14], [15].

Several studies have shown that cyclic strain increases protein synthesis and affects proliferation, migration, and cytoskeletal arrangement in many cells such as endothelial cells [16] and smooth muscle cells [6]. It also delivers anti-apoptotic and proliferative signals to gingival fibroblasts. To the best of our knowledge, there is a dearth of literature regarding the behavior of hPDLCs under the effect of cyclic strain [17]. Therefore, in this study, we provide evidence that cyclic strain promotes cytoskeletal rearrangement of hPDLCs, which gradually arranged parallel to each other, perpendicular to the direction of the tension force, with elongated cell bodies.

How do hPDLCs convert cyclic strain to biochemical signals? A previous proteomic analysis on low shear stress-induced vascular remodeling has demonstrated that Rho-GDIa could respond to shear stress and modulate vascular smooth muscle cell migration and apoptosis [6]. Hence, it was hypothesized that Rho-GDIa might participate in the mechanism via hPDLCs, which sense and transduce the extracellular cyclic strain stimuli. Rho-GDIa is a member of Rho GDP dissociation inhibitors (Rho-GDI), which have been shown to negatively regulate the activities of small G proteins of the Rho family by shutting off their GDP (inactive)/GTP (active) cycling and cytosol (inactive)/membrane (active) translocation [18], [19], [20]. Our findings demonstrated that, with time, physiological cyclic strain downregulated the expression of Rho-GDIa in the experimental group, compared to the static group. Using siRNA-targeted transfection, the decrease in Rho-GDIa expression was accompanied by the enhancement of GTP-Rho, Rock and p-cofilin protein expression. In contrast, with overexpression of Rho-GDIa induced by the plasmid construct, GTP-Rho, Rock and p-cofilin protein expressions were markedly reduced. These results suggest that Rho-GDIa plays a significant role in mechanotransduction and regulation of hPDLC functions in response to cyclic strain.

As we all known, Rho family proteins play an important role in cytoskeletal rearrangement. RhoA is one of the proteins of the Rho GTP family, and its activation can be negatively regulated by Rho-GDIa [6]. Hence, we investigated the activation of RhoA and the reported downstream signaling molecules, such as ROCK, p-cofilin. Contrary to the expression of Rho-GDIa, expressions of GTP-Rho, Rock and p-cofilin were enhanced by physiological cyclic strain, compared with the static control. Therefore, we hypothesized that cyclic strain might modulate the expression of Rho-GDIa and GTP-Rho, Rock and then subsequently regulate the phosphorylation of cofilin. The effect of the specific chemical inhibitor Y-27632 on the hPDLCs in our study appeared to support this hypothesis. The Rock inhibitor reversed the effects of cyclic strain on phospho- cofilin expression as well as cytoskeletal rearrangement.

Migration of hPDLCs is an anchorage-dependent process, and cytoskeletal reorganization is one of the important changes that occur during this process [21], [22], [23], [24]. F-actin is a fundamental component of all eukaryotic cells and provides force as well as stability to the cell [24], [25]. The initial events of cell migration, such as exploration, adhesion, and polarization, require regulated assembly and disassembly of F-actin [26]. Several studies have suggested that Rho family proteins play an important role in cytoskeletal rearrangement [27], [28], [29], but their effect on hPDLCs is still unknown. Immunofluorescence experiments were used to observe the expression of F-actin in hPDLCs subjected to cyclic strain alone compared to those treated with Y-27632 before cyclic strain treatment. The results indicated that it promoted stress fiber formation in hPDCLs in a Rho-dependent manner, leading to orthodontic tooth movement and alveolar bone remodeling.

**Figure 5:**
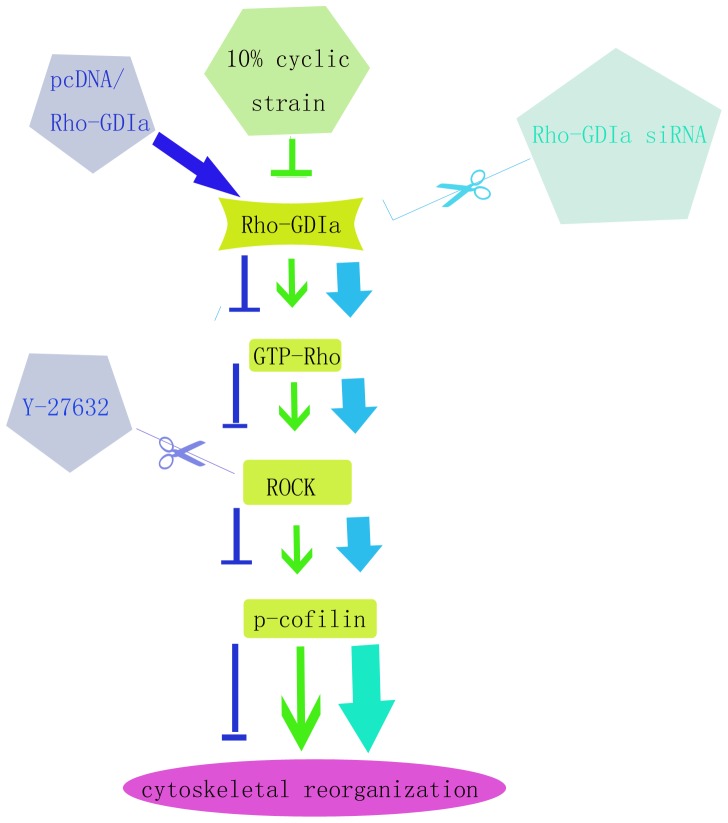
Schematic drawing outlining the pathway of the Rho-GDP dissociation inhibitor alpha, GTP-Rho, Rock and p-cofilin that mediates the enhancement of migration and cytoskeletal reorganization by cyclic strain treatment. Inhibitors and activators of each of the molecules in the pathway regulate the effect of cyclic strain on the member(s) downstream, but not upstream. These studies allow the determination of the hierarchical relationships among the signaling molecules shown in this diagram.

In summary, we show here that cyclic strain reduces Rho-GDIa expression, which upregulates GTP-Rho, which in turn upregulates ROCK, p-cofilin. This mechanism promotes actin polymerization, which may be responsible for cytoskeletal rearrangement (Fig. 5), thus stimulating orthodontic tooth movement and alveolar bone remodeling. Thus, this study may help to further understand the role of these proteins as well as the Rho signaling pathway in the regulation of periodontal tissues while responding to mechanical stimuli.

## Supporting Information

Figure S1 **A: Anti-cytokeratin group were stained negative. B: Anti-vimentin group were stained positive.** (×200).
(TIF)

Figure S2 **Silencing of Rho-GDP dissociation inhibitor alpha on Rho-GDIa protein expression in cultured unstimulated cells.** The protein expression levels of Rho-GDIa in the Rho-GDIa siRNA transfection group were significantly less than those in the negative control (NC) group. Values shown are the mean±SD for each group from three independent experiments. * p<0.05 vs. NC group. All the band intensities are firstly normalized to the loading controls then normalized to the 0h treatment and loading control ratio.
(TIF)

Figure S3 **Overexpression of Rho-GDP dissociation inhibitor alpha on Rho-GDIa protein expression in cultured unstimulated cells.** The protein expression levels of Rho-GDIa in cells overexpression Rho-GDIa group were significantly more than those in the Mock control group. Values shown are the mean±SD for each group from three independent experiments. * p<0.05 vs. Mock group. All the band intensities are firstly normalized to the loading controls then normalized to the 0h treatment and loading control ratio.
(TIF)
