# Supplementary figures and images for: Cyclic Strain-Induced Cytoskeletal Rearrangement of Human Periodontal Ligament Cells via the Rho Signaling Pathway
Source: PLoS One. 2014 Mar 11;9(3):e91580. doi: 10.1371/journal.pone.0091580 (PMC3950223; doi:10.1371/journal.pone.0091580)

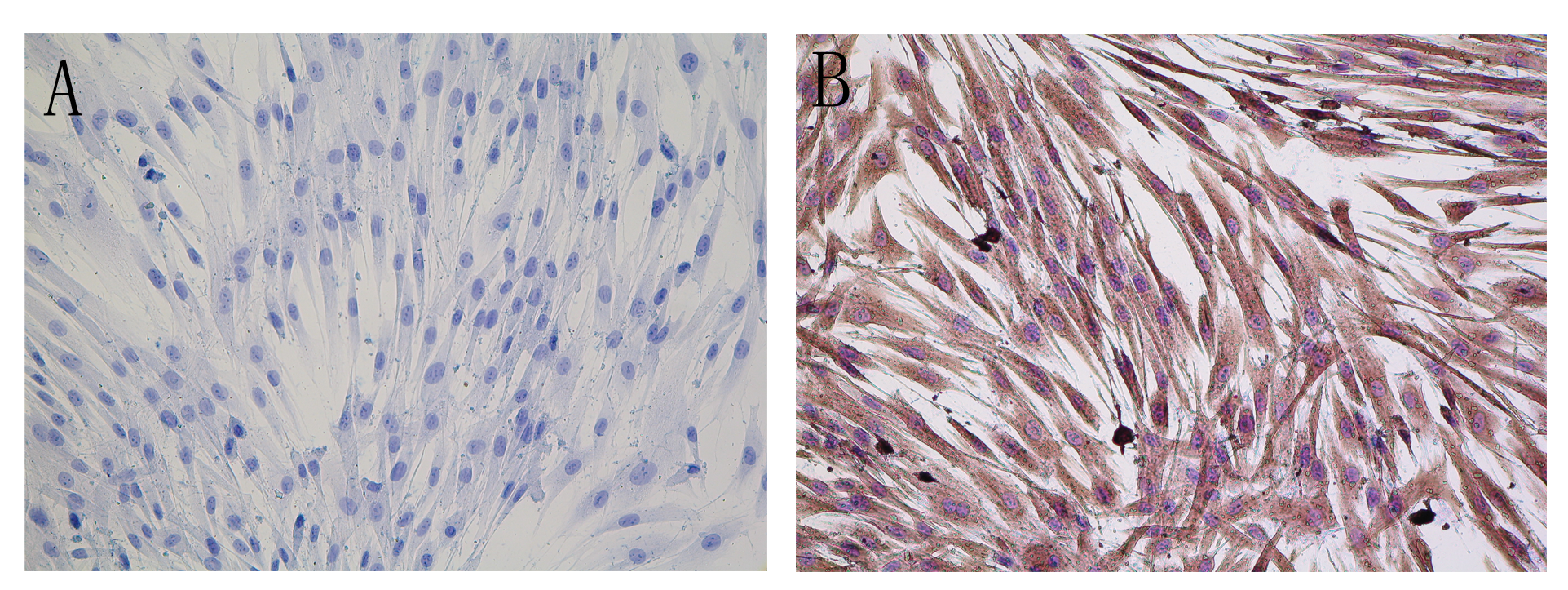

Supplement: Figure S1 — A: Anti-cytokeratin group were stained negative. B: Anti-vimentin group were stained positive. (×200). (TIF) [file pone.0091580.s001.tif]

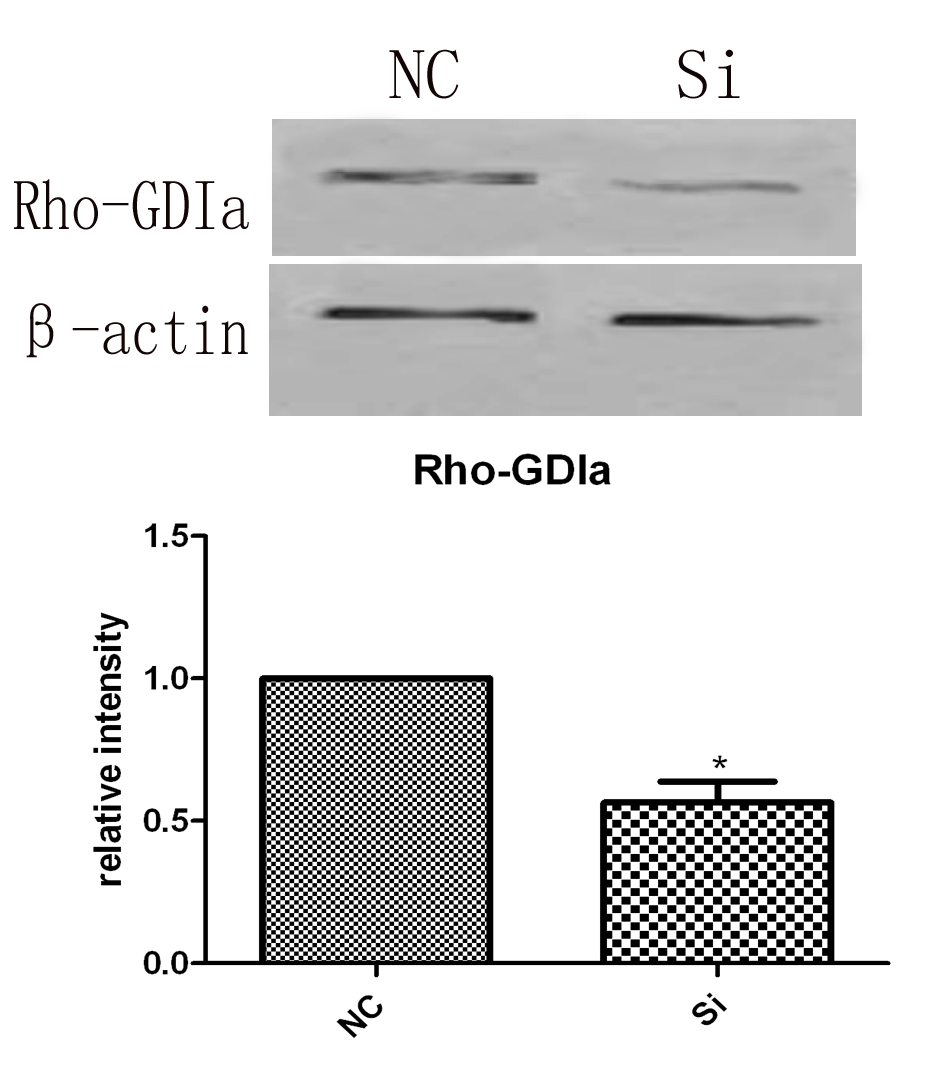

Supplement: Figure S2 — Silencing of Rho-GDP dissociation inhibitor alpha on Rho-GDIa protein expression in cultured unstimulated cells. The protein expression levels of Rho-GDIa in the Rho-GDIa siRNA transfection group were significantly less than those in the negative control (NC) group. Values shown are the mean±SD for each group from three independent experiments. * p<0.05 vs. NC group. All the band intensities are firstly normalized to the loading controls then normalized to the 0h treatment and loading control ratio. (TIF) [file pone.0091580.s002.tif]

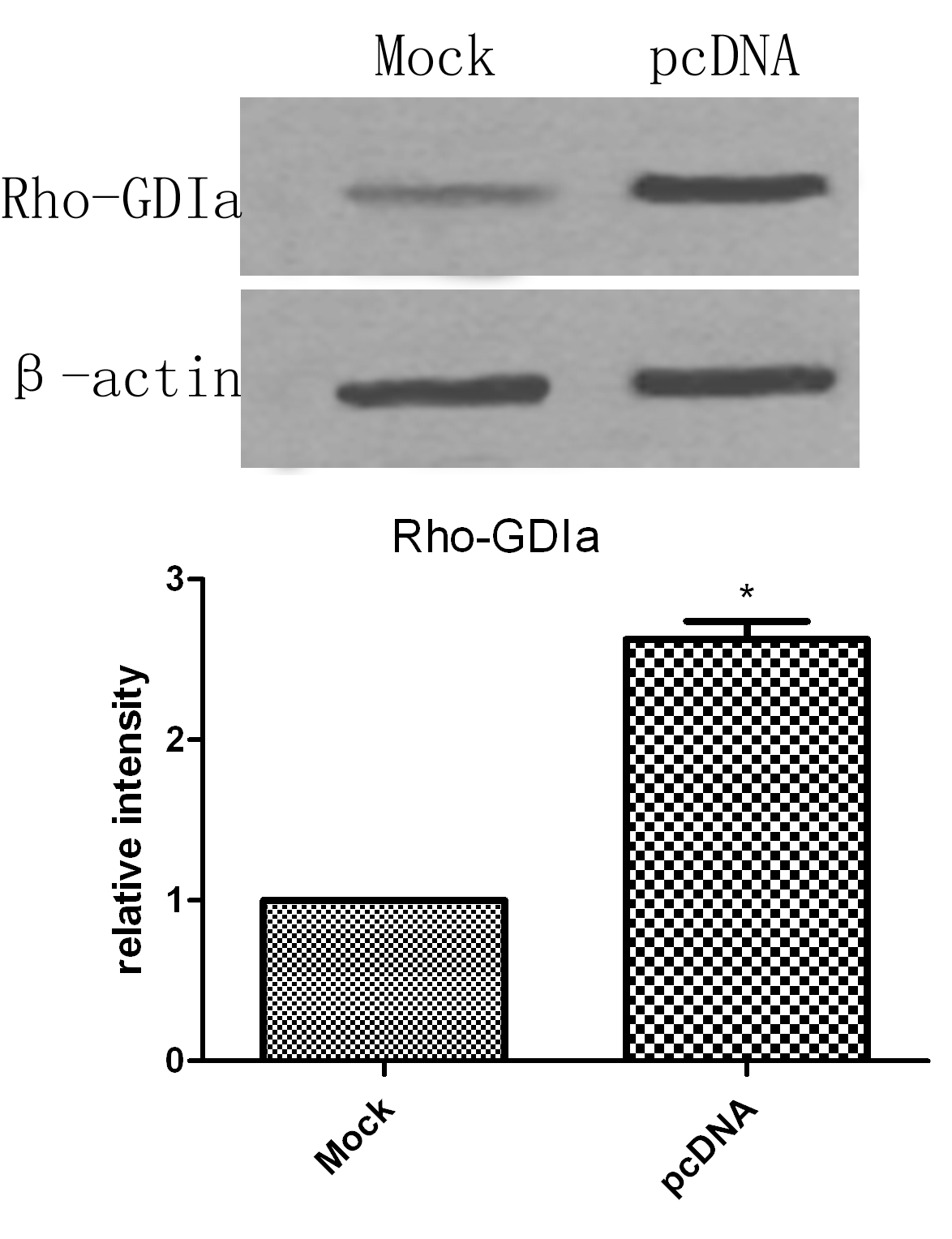

Supplement: Figure S3 — Overexpression of Rho-GDP dissociation inhibitor alpha on Rho-GDIa protein expression in cultured unstimulated cells. The protein expression levels of Rho-GDIa in cells overexpression Rho-GDIa group were significantly more than those in the Mock control group. Values shown are the mean±SD for each group from three independent experiments. * p<0.05 vs. Mock group. All the band intensities are firstly normalized to the loading controls then normalized to the 0h treatment and loading control ratio. (TIF) [file pone.0091580.s003.tif]
